# Supplementary material for: Are carbon emissions trading and green financial instruments synergistic? -Comprehensive quantitative research based on content analysis
Source: PLoS One. 2024 Mar 7;19(3):e0298601. doi: 10.1371/journal.pone.0298601 (PMC10919601; doi:10.1371/journal.pone.0298601)
Supplement: S1 Data — (ZIP) [file pone.0298601.s001.zip › Data/地级市Z_F工作报告.zip!/╔╜╢1⁄2/╝├─■╩╨/└┤╫╘╣1⁄2╓┌║┼í╛┬φ┐╦╩2╛▌═°í┐_2007.docx]

各位代表：

　　现在，我代表济宁市人民政府向大会报告工作，请予审议，并请市政协委员和其他列席会议的同志提出意见。

　　一、“十一五”开局之年经济社会发展取得新成绩

　　过去的一年，在中共济宁市委的坚强领导下，全市各级政府团结带领广大干部群众，全面落实科学发展观，认真执行国家宏观调控政策，全力推进科学发展、加快发展、和谐发展，圆满完成市十四届人大四次会议确定的年度任务目标,实现了“十一五”良好开局。

　　（一）国民经济平稳较快增长。完成地区生产总值1456.1亿元、增长16.4%，三次产业比例由上年的14∶55∶31调整为12.8∶55.2∶32。规模以上固定资产投资491.2亿元、增长17.6%。地方财政收入81.6亿元、增长23.3%，税收收入占比增加到74.9%。新农村建设扎实推进。农业“十大基地”建设进展顺利，粮食总产稳定增长，79个无公害、绿色、有机农产品获得省级以上质量认证，产业化龙头企业发展到552家。防控高致病性禽流感取得阶段性成果。全市规模以上工业企业突破2000家、新增442家，完成增加值607亿元、利税248.4亿元、利润155.1亿元，分别增长27.2%、22.6%和24.1%。高新技术产业产值占比提高到25.6%。新创2个中国名牌、32个山东名牌。全社会消费品零售总额499.6亿元、增长16.3%。知名商业品牌“银座”、“苏果”入驻济宁。首届现代农业及乡村游国际博览会获得圆满成功。全市旅游社会总收入突破100亿元、增长23.4%。民营经济上缴税金79亿元、增长40.1%。举办金融论坛和第二届金融生态环境建设暨银企合作促进会，金融机构年末存贷款余额分别达到1065.7亿元和659.1亿元。

　　（二）改革开放迈出坚实步伐。农村综合配套改革稳步实施。国有粮食购销企业改革基本完成。事业单位改革进展顺利。国有资产监管体系进一步完善。困难企业改制脱困力度加大。太阳纸业股票成功上市。市直91个预算单位先期推行国库集中支付、会计集中核算改革。“促强扶弱”加快县域经济发展的政策措施落实到位，6个欠发达县进入国家奖补扶持范围。新组建济宁市商业银行。煤炭资源有偿使用制度改革试点工作启动。全年新批办外资项目79个，直接利用外资3.15亿美元、增长33.9%。引进市外国内资金169.6亿元。完成外贸进出口23.5亿美元，其中出口15亿美元、增长30%。15个省级园区通过国家核准。济宁高新区实现技工贸收入530亿元、增长31.9%。

　　（三）城乡人居环境持续改善。确立新一轮城市发展战略定位，按照“东拓西跨南联北延”的思路展开济宁城区建设布局。城区河湖水系综合整治全面展开，古运河治理一期工程基本竣工。完成327国道改线北移和红星东路拓宽改造、洸河路西延及车站南路等主干道新建改建任务，滨湖路一期工程开工建设，济宁机场改建加快推进。天然气引进主体工程基本完成。市区新增绿地面积58万平方米、集中供热面积60万平方米。列入国家淮河流域和南水北调计划的重点治污项目加紧实施，9处城市污水处理厂建成运行。累计完成32家燃煤电厂脱硫改造，化工发酵企业异味污染得到有效遏制，城区空气质量优良率达到92.6%。整顿规范土地市场秩序成效明显。治理采煤塌陷地3.1万亩，南四湖人工湿地面积扩大到4.5万亩。新农村建设“321”试点示范工程全面启动。1518个行政村完成“一池三改”，农村自来水普及率达到88.1%。新建乡村公路1500公里。

　　（四）和谐社会建设扎实有效。市政府为民所办10件实事如期完成。全市新增城镇就业8.5万人、其中安置下岗失业人员再就业4.3万人，城镇登记失业率3.3%。农村劳动力转移就业20.8万人。城镇居民人均可支配收入11996元、增长11.7%，农民人均纯收入4590元、增长11.2%。各项社会保险参保人数与基金征缴同步增长，适当调高并兑现部分社保补助标准。失地农民基本生活保障制度初步建立，农村“五保”集中供养率提高到71.8%。市县财政投入低保资金7219.6万元，4.1万户城乡低保对象基本生活得到保障。济宁市区新建经济适用房10.2万平方米，348个“双困户”住上廉租房。改造农村中小学危房15.4万平方米，彻底解决部分学生带桌凳上学问题，救助困难学生7万名。慈善机构和红十字会在扶贫济困中发挥积极作用。“平安济宁”建设深入推进，社会治安防控体系更加严密，信访责任制进一步落实，安全生产形势持续稳定好转，微山湖地区鲁苏共建和谐边界工作经验在全国推广。

　　（五）各项社会事业协调发展。成功举办第七届中国专利高新技术产品博览会和“院士济宁行”活动，全年完成重要科技成果166项，申报专利1541件、授权824件，建成国家级纺织新材料产业基地，新增省级、国家级工程技术中心11家、省级以上高新技术企业28家。实施教育优先战略，市县财政性教育投入明显增加，“双高普九”成果巩固发展，省级规范化学校增加到73所。实施市属普通中专布局调整和资源整合，组建起新的山东贸易职工大学。济宁师专成功晋升济宁学院。确定为教师所办8件实事正在逐一落实。国际孔子文化节综合效应持续提升，颁发首届联合国教科文组织“孔子教育奖”。中华文化标志城前期工作有新进展。3个非物质文化遗产项目入选首批国家级保护名录。36处中心镇卫生院改造基本完成，新型农村合作医疗试点在全市推开。城市社区卫生服务网络进一步健全。第二次农业普查全面展开。工商、物价执法管理措施加强，食品、药品市场监管体系逐步完善。人口自然增长率控制在5.24‰。我市体育健儿在21届省运会上取得好成绩。经济社会信息化进程加快。新闻出版、广播电视、民族宗教、外事侨务、人防地震、水文气象、档案史志、老龄和残疾人等事业获得新发展。

　　（六）民主法制和精神文明建设得到加强。自觉接受人大及其常委会法律监督和政协民主监督，全年办理人大代表建议180件、政协提案345件。颁布依法行政“四五”规划，清理公布市级行政许可事项535项，启动实施 “五五普法”。政务公开制度日益完善，行政审批服务水平不断提升，审计监督和行政效能监察力度加大，反腐倡廉取得新的成果，商业贿赂专项治理和纠正部门行业不正之风成效明显。“市长公开电话”运转灵敏高效，群众反映的大量实际问题得到及时解决。“文明济宁”建设深入展开，15个单位获得全国文明创建先进单位称号。国防动员教育、民兵预备役建设和双拥共建富有成效。

　　各位代表，过去一年取得的成绩，是省委、省政府亲切关怀、正确领导的结果，是市委统揽全局、科学决策的结果，是市人大、市政协有效监督、大力支持的结果，是全市人民同心同德、拼搏实干的结果。在此，我代表市人民政府，向全市工人、农民、知识分子和干部，向各民主党派、工商联、人民团体和各界人士，向中国人民解放军驻济部队指战员、武警官兵和公安干警，向中央、省驻济单位，向来济宁创业发展的投资者和建设者，向关心支持济宁现代化建设的港澳台同胞、海外侨胞和国际友人表示崇高的敬意和衷心的感谢！

　　我们也清醒地认识到，经济社会发展中还存在不少困难和问题，政府工作也有许多需要改进的地方。制约经济发展的结构性、机制性矛盾仍较突出，主导产业层次提升缓慢，新兴服务业、高新技术产业比重偏小，经济外向度不高；农业产业化经营水平低，农村公共设施建设相对滞后；县域经济发展不平衡，部分县乡财政还比较困难；城市建设重点项目推进不快，中心城区辐射带动功能尚未充分显现；自主创新能力不强，节能降耗、污染减排形势依然严峻；就业、社保面临较大压力，影响社会和谐稳定的因素仍然不少；政府职能转变不快，思想观念、领导方法、工作模式还不能完全适应新形势新任务要求，有的地方和部门作风不实、效率不高、服务不优，影响发展环境的问题时有发生。对此，我们一定要高度重视，认真解决。

　　二、新一年工作的总体要求和任务目标

　　2007年是本届政府任期的最后一年。做好今年各项工作，圆满完成任务目标，意义重大，影响深远。根据省委、省政府和市委统一部署，今年政府工作总体要求是：以邓小平理论和“三个代表”重要思想为指导，以科学发展观为统领，以扩大开放、深化改革为动力，围绕建设经济强市、文化名市、组群结构大城市的奋斗目标，按照实施“四大战略”、抓好“三个关键”、突出“五大重点”、实现“六个显著提升”的总体思路，大力实施项目带动，强化“三强一增一促”，构建完善金融服务、现代物流、科技创新和人力资源开发、发展环境优化“五大支撑体系”，突出抓好新农村建设、主导产业培植、节能降耗减排和城市规划建设管理等项重点工作，更加注重改善民生促进社会和谐，切实转变政府职能改进工作作风，开创经济社会又好又快发展新局面。年度主要预期目标是，地区生产总值增长14%，地方财政收入增长17%，全社会固定资产投资增长25%，外贸进出口和出口均增长20%，直接利用外资增长20%，引进市外国内资金增长25%，社会消费品零售总额增长16%，城镇居民人均可支配收入和农民人均纯收入均增长8%,城镇登记失业率控制在4%以内。主要约束目标为，万元生产总值能耗降低4.7%，二氧化硫排放减少8%，化学需氧量排放降低6.2%，人口自然增长率控制在6.7‰以内。

　　完成今年任务目标，我们面临诸多有利条件。世界经济增长态势持续向好，国际和地区间产业资本转移步伐加快；国家科学发展的政策导向更加明确，扩大内需的拉动力不断增强；我市煤电水及人文资源优势明显，众多骨干企业加速扩张发展，一批产业项目陆续建成投产；调整后的各级领导班子面貌一新、坚强有力，干事创业的热情空前高涨。这些都坚定了我们在新起点上推进又好又快发展的信念和决心。同时也要看到，国际经济环境的不确定因素，人民币汇率的变动，项目建设准入门槛的抬高，节能环保刚性约束的增强，加之区域竞争更趋激烈，又给我们带来许多新情况、新问题。面对前所未有的机遇和挑战，我们务必增强责任感、紧迫感和危机感，坚决做到加快发展不动摇，让我市经济实力一天比一天强；坚决做到真抓实干不松劲，让我市城乡面貌一天比一天新；坚决做到改善民生不懈怠，让我市老百姓日子一天比一天好！

　　三、全力推动经济又好又快发展

　　做好今年经济工作，必须以调整优化结构、转变增长方式为着力点，集中在解决关系发展全局的重大问题上强化措施、务求实效。

　　（一）加快发展现代农业，扎实推进新农村建设。坚持把解决“三农”问题作为全部工作的重中之重，把发展现代农业作为“三农”工作的首要任务，把增加农民收入作为“三农”工作的最终目标，加大倾斜扶持，解决实际问题，促进农业增效、农民增收、农村和谐发展。

　　深度调整农业结构。细化分解十大优势特色产业年度建设目标，集中扩张发展100个规模化、标准化种养基地，扶持创建农产品质量安全示范区。突出发展高档瓜菜、苗木花卉、甜叶菊为主体的城郊型产业基地，优质专用粮、棉、油和畜禽养殖基地，优质果品和速生林基地以及精品渔业、休闲渔业基地，培育更多专业镇村和专业户。全市高价值经济作物扩大到400万亩，争取再创10个无公害、绿色、有机农产品知名品牌。

　　提升产业化经营水平。建立种养结构调整与产业化龙头项目建设的良性互动机制，发挥好专项资金的吸附引导作用，支持现有龙头企业实施扩能改造，鼓励外资、民资和城市工业投资农副产品精深加工。注重培育发展产地市场，面向国内大中城市开辟农产品营销“绿色通道”。全年新增龙头企业100家，扶持100家农村合作经济组织和专业协会，带动450万亩种养基地、100万农户进入产业化经营体系。

　　改善生产装备条件。实施沃土工程，改造中低产田40万亩。抓好南四湖湖东堤修筑、湖西大堤加固等大型水利工程，争取开工泗河、东鱼河二期等骨干河道治理项目，完成西苇、贺庄等大中水库除险加固任务，加快推进去冬今春1.3万项农田水利工程。及早实施南水北调系列项目。坚持增加农机总量与优化装备结构并重，年内力争农业机械化综合水平达到70%以上。办好济宁农业高新技术示范园和各类农业科技园区。加快农产品质量标准和检验监测体系建设，完善动植物疫病防控网络。

　　强化支农惠农政策措施。今年集中为农民群众解决好15个方面的实际问题。一是健全农村投入稳定增长机制，市级财政安排“三农”投入3.2亿元，比上年增加8000万元。二是市财政安排专项资金2000万元，用于全部免除农村义务教育阶段学生杂费，继续对农村贫困家庭学生免费提供课本并补助寄宿生生活费。三是市财政投入500万元，改造农村中小学校舍危房5万平方米。四是建立完善农村最低生活保障制度，保障标准每人每年不低于800元。五是年内农村新型合作医疗制度覆盖所有乡镇，市财政配套资金4071万元，比上年增加1955万元，补助标准由每人每年30元提高到40元。六是市财政投入1150万元实施助残济困“三个一千”工程，为1000户包括残疾人在内的农村贫困家庭改造危旧住房，为1000名贫困肢体残疾人配发轮椅，为1000名贫困白内障患者实施复明手术。七是市财政配套4931万元，新建改造农村公路1000公里，并建立农村公路养护管理机制。八是市财政拿出2000万元，完成523个村41.4万人的供水工程项目建设，全市农村自来水普及率达到95%以上。九是全面实施城乡用电同网同价，减轻农民负担8100万元。十是市财政投资600万元，再对10%的行政村实施“一池三改”，发展5万户沼气池。十一是力争45%的行政村通有线电视。十二是资助40个经济薄弱乡镇建设文化中心站、100个村建设文化大院，为2000个村配备图书60万册。十三是积极探索农村房产、土地经营权和相关收益权抵押贷款担保方式，持续扩大农户小额贷款发放规模，选择部分镇村先期推行政策性农业保险。十四是组织3000名科技特派员深入生产一线，集中推广农业先进实用技术和优良品种。十五是继续实施百万农民培训工程，年内新增农村劳动力转移就业20万人。对这些事项，我们要逐一明确责任，落实资金，加紧实施，确保兑现，真正让公共财政的阳光普照农村大地，让改革发展的成果惠及广大人民群众！

　　（二）大力实施项目带动，培育壮大优势产业。牢固确立“工业立市”理念，立足做大做强做优“五大工业集群”、“四大服务产业”，依据国家产业政策导向，抓紧抓好“三个一批”，靠“高大外”项目的引进建设带动投资结构优化、产业层次提升、骨干企业膨胀。

　　围绕提高主导产业规模效益抓项目。紧扣煤炭精深加工、机械制造产业链拉长、生物技术主导产品扩张、医药食品新产品开发、纺织服装名优品牌培育的年度目标，集中推进科技含量高、产出效益好、关联带动作用强的产业龙头项目，突出抓好国宏甲醇、小松行走系统、重汽商用车扩能、正大菱花赖氨酸、雪花味精、如意精纺呢绒等一批亿元以上大项目。做好华能济宁电厂、微山华润电厂等电力骨干项目前期工作。年内争取将我市规划定点为国家级煤化工基地。支持鼓励县市区发挥比较优势，选准主攻方向，着力突破一批先进制造业大项目。今年全市新开工投资过亿元项目100个以上。

　　围绕壮大骨干企业群体抓项目。加紧推进360个投资超千万元的重点产业项目，力争一步到位做成大企业。实施“优势工业产品膨胀计划”，年内销售收入过5亿元的单个产品突破30个。抓好太阳纸业30万吨无菌液态包装纸、易路400万套轿车子午胎、如意天容20万纱锭紧密纺和泰山玻纤新材料、联诚金属三期等一批技改扩能、新产品开发和资本运营、合资合作项目，促使优势企业尽快晋档升级。支持驻济大企业提升产业层次和规模效益，更好地引领支撑区域经济发展。今年全市利税超千万元企业突破200家、新增40家，6家重点企业销售收入过50亿元，山推集团、太阳纸业力争率先实现销售收入过百亿目标。

　　围绕培植新兴服务业增长点抓项目。旅游产业，深入实施《济宁市旅游发展总体规划》，集中抓好尼山、微山岛、南阳古镇、宝相寺、泗水泉林、水泊梁山等一批景区景点开发建设，做精观光农业游、生态休闲游、民俗风情游等新兴旅游项目，整合推出几条精品线路，加紧建设游客服务中心等配套设施，开发健康、文明、参与性强的娱乐项目。年内全市接待中外游客突破1700万人次，实现旅游社会收入120亿元以上。文化产业，结合城区古运河沿岸开发建设，打造特色文化产业聚集带，加紧引进落实动漫、广告、形象设计等一批创意文化项目，探索报业、出版、广电、演艺企业化运营、产业化发展新路子。物流产业，依托交通货运场站、海关保税区及重点园区，布局建设黄屯、华能陆港等大型物流中心；支持骨干企业创办第三方物流项目，集中扶持安海、金宇等重点物流企业率先成长壮大；高起点规划建设现代化港口、码头及仓储服务区等配套设施，打响“运河物流”品牌。继续抓好传统商贸流通业改造提升，争取再有几个国内外知名品牌和商贸集团入驻我市。结合实施“万村千乡”市场工程，城乡一体发展超市、连锁、配送等新型流通业态，办好村级综合服务中心。金融保险业，支持各商业银行深化改革、创新服务。做大做强圣泰农村合作银行、济宁市商业银行等地方金融机构。鼓励各类资本到农村设立多种形式的贷款机构。积极引进市外、境外金融企业来我市设立分支机构。拓展保险业经营领域，做好法定保险的实施和大众责任保险的推行工作。继续兴办科技研发、中介咨询、就业服务等新兴服务业项目。今年全市服务业增加值占GDP的比重提高1-2个百分点。

　　围绕项目建设强化保障服务。一是依法完备手续，规范建设行为。所有项目都要符合国家产业政策，依法完善立项、用地、环评及相关手续。二是节约集约用地，满足建设需要。严格依照土地利用总体规划布局项目建设，落实耕地保护目标责任制，坚决杜绝违法用地。多方盘活存量土地，充分利用闲置厂房、滩涂坡地以及村庄规划整理腾出的土地。引导各类园区压减公共建设用地，发展多层厂房，提高单位面积投资强度。积极争取、用足用好政策，千方百计保证重点工程、重大项目、鼓励支持类产业项目建设需要。三是搭建融资平台，密切银企合作。做实做大市县两级投融资平台，分层次扶持组建一批担保机构，继续开展信用乡镇、社区、企业、农户评选活动。建立完善政府、银行、企业有机对接、良性互动的金融合作平台与服务机制，筹备办好今年银企合作促进会。各类金融机构要优化信贷结构，集中资金投向重点项目、骨干企业和成长性好的中小企业，大幅提高存贷比。四是落实包保责任，优化建设环境。把各类建设项目尤其是投资500万元以上产业项目纳入目录管理，逐一明确相关责任人及具体包保措施，亿元以上大项目继续实行“一个项目一名领导包保、一套班子运作、一条龙服务”责任制。有关部门要坚持严格审核把关与优质高效服务相统一，确保项目建设顺利推进。

　　（三）发展壮大县域经济，不断增强综合实力。深入实施促强扶弱战略，改进完善县市区主要经济指标及“强园区强乡镇强企业”发展考核办法，促使经济强市放大领先优势，中间县市尽快进位升级，欠发达县加快赶超步伐。把振兴工业作为主攻方向。持续增加有效投入，大上先进制造业、高新技术产业、资源精深加工等项目；引导支持现有企业联强联外联高，尽快膨胀规模实力。今年各县市区新增规模以上企业突破50家。把民营经济作为主导力量。用好民营企业发展专项资金，实施“中小企业成长计划”，支持民营大户实施低成本扩张、多元化经营，吸引国内知名民营企业来我市投资兴业。今年全市民营经济增加值和上缴税金分别增长25%、30%以上。把培植“三强”作为关键抓手。突出抓好省级园区基础设施配套，研究制定吸引力强的政策措施，吸引更多资金投向园区，“高大外”项目落户园区，扩张发展的骨干企业聚集园区。健全“强乡镇”动态管理机制，引导乡镇大上产业项目，发展壮大村级集体经济。深入实施“梯次推进”战略，促使骨干企业大搞合资合作和技术改造，现有规模以上企业持续扩张生产能力，培植更多立县立乡强企业。把增加可用财力作为重要目标。找准国家产业政策、财税政策与县乡基础优势的结合点，集中兴办税收贡献大、地方留成多的产业项目。着力强化税收征管，完善综合治税措施，严格杜绝税源流失，提升“两税”收入比重。优化财政支出结构，用好转移支付资金，保障重点支出需要。有效化解乡村债务，杜绝新债发生。

　　（四）努力扩大开放深化改革，进一步激发发展动力与活力。坚持抓开放抓招商不动摇。着眼国外境外招商与国内招商两个成果一起要，筹划组织好几次大型招商活动。国外境外招商主攻日韩、欧美、港澳、台湾，精心包装、集中推介、专案突破一批重点招商项目。国内招商以长三角、珠三角和京津地区为重点，同步加强与周边地区的联合协作，力争在引进市外国有大集团、民营大户和台资企业方面取得大的进展。强化省级园区招商载体功能，突出抓好主导产业项目专题招商、上下游产业链招商、优势企业股权招商和现有外资企业以商招商。济宁高新区集中引进基地型、龙头型、高端型外资大项目，加速形成以煤化工、工程机械、生物医药、纺织新材料等产业为主体的外资企业集群。发挥企业招商主体作用，督促骨干企业尤其是利税超千万元企业展开“一对一”洽谈，尽快办成合资合作大项目。巩固扩大多领域招商成果，着重在城市公用设施、农业产业化、金融保险、商贸物流及文化旅游产业等领域突破一批外资项目。更加重视搭建并用好各种招商平台，务求今年曲阜国际孔子文化节取得更为明显的招商成果。今年各县市区都要办成2－3个投资超千万美元的外资大项目，曲阜、兖州、邹城3市直接利用外资都要突破8000万美元，济宁高新区突破1亿美元。调整优化进出口商品结构，扩大农副产品精深加工、机电产品和高新技术产品出口规模，优先进口重要原材料和急需先进设备、关键零部件。支持有条件的企业境外投资办厂、开展工程承包和劳务合作。

　　扎实推进各项改革。指导帮助骨干企业搞好规范化股份制改造，培植更多上市后备企业。逐一制定落实市属困难企业改制脱困方案，争取一批拟破产企业及早进入破产程序。完善国有资产监管体系，建立经营预算制度，严格经营业绩考核，确保国有资产保值增值。稳步实施财政体制改革，加快推行部门预算，规范完善“收支两条线”制度，扩大政府采购范围和规模，尽快将市直所有预算单位纳入财政集中支付、会计集中核算运作体系。深化农村综合配套改革，进一步精简乡镇机构，有效整合事业站所，加快推行“乡财县管乡用”。

　　（五）强化科技进步与创新，增创竞争发展新优势。突出企业在科技进步与创新中的主体地位。支持骨干企业高起点建设研发机构，今年再建成5个省级以上工程技术中心。督促企业摊够、提足、用好技术开发费用，扶持发展120家民营科技企业。抓好科技成果转化应用和二次创新“两个重点”。引导各类企业积极吸收应用最新科技成果，加大传统产业信息化改造力度，促使电子信息等新兴产业成长壮大。有效利用市内外高等院校、科研院所的研发力量，集中开展联合攻关和引进消化吸收再创新，力争在煤化工、机械制造、生物医药、纺织新材料等领域突破一批共性、关键和核心技术。强化知识产权保护管理。依托鲁南质检中心，争取建设国家级检验实验室和检测中心。年内取得重要科技成果160项，专利申请量增长30%，争创20个以上省级、国家级名牌产品，开发高新技术产品350个，新增高新技术企业60家，高新技术产业产值比重再提高3个百分点。强化产学研结合、科技服务平台和高层次人才“三个保障”。办好今年中国专利高新技术产品博览会和“院士济宁行”活动，新建一批中试基地和博士后工作站。完善市县生产力促进中心运行机制，支持重点专业孵化器扩充面积、提升功能。探索建立创业风险投资和技术交易市场。依托驻济高校和各类职业技术院校大力培养实用型技能人才。面向海内外选聘高层次专门人才，启动实施“百名博士引进工程”。重视引进国外智力。对创新创造创业贡献突出的优秀人才给予重奖。

　　（六）着力抓好节能减排，确保完成年度控制目标。狠抓重点行业和用能大户节能降耗。逐一落实煤炭、电力、化工、建材、造纸、生物医药等6个重点行业和100家重点耗能企业的节能降耗年度控制目标，推广应用50项节能新技术新成果，限期淘汰耗能高、污染重的落后生产工艺装备。督促企业落实生产全过程成本控制措施，搞好重点耗能单位节能计量监察和能源审计，执行超标用能惩戒和节能奖励制度。严格控制新上高耗能产业项目。加快重点治污项目建设进度。督促211家重点废水排放企业依照省定标准，细化落实深度治理措施，确保所有点源治理项目7月底前全部建成投运。完成40家企业清洁生产审核，抓好10个“三废”资源化项目，集中培植4家循环经济示范园区和10家先导示范企业。加快推进城市污水处理厂管网配套，认真执行污水处理收费新标准，确保所有城市污水处理厂正常运行、出水水质达标。年内建成济宁生活垃圾处理厂。争取7处截污导流工程及早开工。巩固扩大燃煤电厂脱硫改造和化工发酵企业异味污染治理成果，全年空气环境质量优良率保持在90%以上。严格执行新上项目“三同时”制度，强化排污总量控制和排污许可证管理。完善环保在线监测平台功能，严厉查处偷排偷放、超标排污行为。强化生态保护与重建。收足用好矿山地质环境治理保证金，探索科学合理的多种治理模式，今年完成3万亩采煤塌陷地治理任务。落实各类生态功能保护区建设管理措施，新增南四湖人工湿地面积2.3万亩。加快荒山、荒滩和沿湖、沿路造林绿化，年内林木覆盖率提高到25%。

　　（七）拉开城市建设布局框架，提升承载能力和品位形象。依据“孔孟之乡、运河之都、水城风貌、生态宜居城市”新一轮发展定位，以中心城区建设为突破口，强力推进“东拓西跨南联北延”，力争城市规划建设多出亮点、上新水平。

　　强化城市规划先导引领作用。组织编制京杭运河沿岸概念性规划，及早完成济宁城区城市景观风貌规划，加快编制京沪高铁曲阜站片区等重点区域控制性详细规划，做好一批年度重点项目修建性规划，严格公路沿线、重点景区及城乡结合部等区域规划控制，依法开展集中拆除规划区内违法建筑专项行动。

　　加快推进城市重点工程项目建设。建立政府统筹规划、建设部门主抓、业主全面负责、相关部门联动的项目建设工作机制，拓宽城市建设投融资渠道，更多地利用市场化手段筹集资金，兴建重大工程项目。把拉开中心城区布局框架作为今年城市建设的核心任务，以建设太白东路跨河大桥、洸府河南段整治开发带动“东拓”；以太白西路跨河大桥、滨湖路建设带动“西跨”；以北湖综合开发和北湖中路、环城西路、柳行路延伸及重点项目建设带动“南联”；以琵琶山路、共青团路、济安桥路、古槐路贯通新327国道和城北区及老327沿线整体开发带动“北延”。全线展开城区河湖水系综合整治，同步实施沿河桥梁翻建改造及河道照明夜景工程。完成7条主干道和背街小巷改扩建任务，升级改造人民公园，高标准一体化规划改造百花公园和儿童乐园，并免费向市民开放。加紧建设国际会议中心，开工建设市体育中心、图书馆、工人文化宫、市民健身广场及金融大厦、报业大厦、创新大厦。积极构建济宁城区至各县市“大公交”网络。确保济菏高速济宁段建成通车，及早完成济宁机场航站区等基础设施建设任务。着眼实现与中心城区的有效对接、融合互动，指导县市区推进城市规划建设。

　　建立城市管理长效机制。深入落实理顺城市管理体制的具体措施，充分发挥街道、社区的作用，全面推行网格化管理和居民自我管理。加强城市综合执法，持续开展环境整治，逐一制定落实城市出入口整治改造方案。科学调控管理城市交通，查禁取缔各种非法运营车辆，提高城市车辆通畅水平。

　　创新城市经营思路办法。完善城市土地收购、储备、出让机制，严格执行经营性用地先详规再招拍挂制度。提升城市建设市场开放度，吸引实力强、资质高、信誉好的大企业、大财团参与我市城市建设。加快推进城市公用事业改制转机，探索重大基础设施建设管理市场化、产业化新模式，公开招标拍卖公用设施的经营权、冠名权，多渠道增加城市经营收益。

　　四、切实改善民生促进社会和谐

　　坚持以人为本、利民为先，财政投入、政策扶持和公共服务进一步向改善民生倾斜，今年市财政预算安排2.7亿元，增长44.8%。继续为民办好10件实事，切实解决好新农村建设中15个方面的实际问题，让城乡百姓亲身感受到和谐社会建设的成效。

　　努力扩大就业。落实就业再就业扶持政策，大力发展就业容量大的劳动密集型产业，重点帮助下岗失业和关闭破产企业人员实现再就业，力争现有“零就业”家庭每户至少1人就业，年内全市新增城镇就业8.5万人。统筹做好高校毕业生、农民工、复员转业军人的就业工作。全面实行劳动合同制度和工资集体协商制度，严格执行最低工资标准，强化劳动监察和争议调处仲裁，构建和谐劳动关系。

　　健全社保体系。今年市级财政安排社会保障支出1.7亿元，比上年增加2055万元。继续扩大社会保险覆盖面，突出抓好外资、民营企业和城镇灵活就业人员的参保工作，切实提高基金征缴率，严格监管制度，确保社保基金安全和保值增值。完善城乡低保动态管理机制，巩固应保尽保，推进分类施保。兑现落实征地补偿标准和被征地农民基本生活保障措施。稳定提高农村“五保”集中供养率。继续加大扶贫力度，重视做好水库移民后期扶持工作。整合捐赠救助资源，健全灾害应急救援体系，积极发展社会福利和慈善事业。加强改进法律援助工作。更多地关注少数民族集居区的经济社会发展。

　　改善住房条件。在全市城乡实施“安居惠民工程”，强化市中区、任城区和济宁高新区的组织实施责任，加快“城中村”、棚户区改造步伐。加紧编制22个中心镇和200个中心村建设规划，结合村庄整理和压煤搬迁，抓好一批新农村建设示范村和小康农户住宅样板工程。加大经济适用房开发建设力度，全面推行廉租房制度。管好用好住房公积金。创新机制，完善政策，加强成本控制和市场监管，下决心抑制并逐步降低普通住房价格水平，努力实现居者有其屋，让老百姓买得起、住得安！

　　实施教育优先。市财政预算安排教育支出3.87亿元、增长14.1%。扎实推进素质教育，深入实施“双高普九”。完善农村义务教育经费保障机制，确保县乡教师工资县域内统一标准发放。促进教育均衡发展，创建省级规范化中小学校30所，高中阶段入学率提高到80%以上。重视学前教育。继续落实贫困生救助措施。加紧实施济宁城区4平方公里教育功能区的选址和规划建设。深入推进职业教育布局调整和资源整合。完善济宁学院基础设施和学科设置。为曲阜师范大学、济宁医学院立足济宁加快发展营造良好环境。鼓励引导民办教育健康发展。

　　强化医疗服务。市财政安排医疗卫生事业支出9856万元、增长14%。着眼健全城市社区卫生服务网络，集中建设30个示范服务中心。对99家乡镇卫生院实施改造，配套建设村卫生室。推行城镇居民大病统筹医疗保险，设立市县两级困难群众大病救助基金。探索建立病种付费限价制度，创办更多“惠民病房”。加强传染性疾病和地方病、职业病防治，提高重大疾病预防控制和突发公共卫生事件救治能力。扶持中医药事业发展。

　　创建精神文明。把社会主义核心价值体系融入文明城市建设全过程，倡树社会主义荣辱观，深入开展群众性文明创建活动。抓好文化信息资源共享工程建设，强化文化市场监管，繁荣文学艺术创作。重视发展哲学社会科学，完善文博、史志、档案社会服务功能。有效推进中华文化标志城前期工作。落实优抚安置政策，加强国防动员教育、人民防空和民兵预备役建设，争创全国双拥模范城“五连冠”。广泛开展全民健身活动，提高竞技体育水平。统筹解决人口问题，持续稳定低生育水平。保障妇女儿童和残疾人权益。重视做好民族宗教、外事侨务、对台和老龄工作。提高气象、地震监测预报水平，增强防灾减灾能力。

　　维护社会稳定。深入落实“平安济宁”建设各项措施，着力强化基层基础。抓好“五五”普法宣传教育。坚持警力下沉，加强社会面治安防控，保持对各种违法犯罪的严打高压态势，切实增强人民群众安全感。深入做好矛盾纠纷排查调处工作，严格落实信访责任制，及时解决群众反映的实际问题。加强社会应急管理。强化企业主体责任，坚持不懈开展专项整治，加大依法监管力度，确保安全生产形势稳定好转。

　　五、努力提高政府自身建设水平

　　面对新形势新任务，各级政府和广大公务员必须切实加强思想政治建设和作风建设，自觉践行胡锦涛总书记提出的八个方面的良好风气，不断提高决策力、理解力、执行力、创造力，为完成全年任务目标、实现又好又快发展提供可靠保障。

　　坚持勤奋学习。深入学习邓小平理论、“三个代表”重要思想以及科学发展观、构建和谐社会等一系列重要论述，认真钻研现代经济、科学技术、社会管理和法律法规等方面理论知识，注重向群众学习、向实践学习。以学习提高政治素养，始终坚持在大局下行动，自觉在思想上、政治上、行动上同上级党委、政府保持高度一致，确保各项方针政策和重大决策部署在政府工作中落实到位；以学习促进思想解放，善于运用新理念、新方略谋划新思路、新办法，破解新矛盾、新问题，牢牢把握发展主动权；以学习强化民本意识，切实把政府追求的目标与老百姓的期盼一致起来，把政府考核的指标与老百姓的评价结合起来，把对上负责与对下负责统一起来，真正做到守土有责、造福一方！

　　坚持改革创新。进一步深化“三学三创”活动，彻底破除“三低三小”，努力实现思想境界、工作标准和办事效率“三个提升”。加快政府职能转变，认真落实创新发展环境二十条规定，突出强化社会管理和公共服务，更好地运用经济、法律手段服务市场主体、管理社会事务、调节利益关系、解决复杂问题。扎实推进行政管理体制改革，办好市县两级行政审批中心，年内完成行政支持类事业单位改革任务。规范政务公开的内容与形式，加强电子政务建设，落实新闻发布制度，办好市长公开电话。从创新体制机制入手，构建新时期群众工作新格局，保障人民群众对政府工作的知情权、参与权、表达权和监督权。

　　坚持依法行政。自觉接受人大及其常委会的法律监督，认真执行各项决议决定，支持人民政协履行政治协商、民主监督和参政议政职能，认真办理人大代表建议、政协提案。严格执行民主集中制，切实维护领导班子的团结。建立完善科学民主决策机制，坚持重大事项集体决策、专家咨询、社会公示与听证、决策评估等项制度。全面落实依法行政“四五”规划，严格依照法律规定的权限和程序行使权力、履行职责。抓好行政执法队伍建设，健全权责明确、行为规范、监督有效、保障有力的行政执法体制，确保公正、文明、严格执法。抓好工商行政管理和市场物价监管工作。加强食品药品安全质量监督。提高统计质量水平。

　　坚持务实高效。大兴求真务实之风，练就抓落实的过硬本领。重大决策、重点工作逐一明确目标责任，细化落实措施，严格时限要求，确保如期完成。从市政府一班人做起，力戒心浮气躁，改进会风文风，摆脱繁琐事务和不必要的应酬，带头深入改革建设一线，重大事项领衔挂帅、亲历亲为，困难大、矛盾多的工作靠上督促整改、限期扭转局面。倡导雷厉风行、只争朝夕的精神，做到议必决、决必行、行必果。完善政务服务流程，健全绩效考核指标体系，推行行政问责制，创建高绩效机关。

　　坚持从严治政。树立正确的世界观、人生观、价值观和地位观、权力观、利益观，筑牢拒腐防变的思想道德防线。强化领导干部廉洁自律，严格执行中央和省、市委反腐倡廉各项纪律规定。全面落实党风廉政建设责任制，加强对领导班子、领导干部的教育、管理和监督。完善源头治腐各项规章制度，健全行政权力监督制约机制，重视新闻舆论监督和社会公众监督，切实强化审计监督。支持纪检监察机关依法查办案件，继续开展治理商业贿赂专项工作，切实纠正侵害群众利益的不正之风。教育引导广大公务员牢记“两个务必”，发扬艰苦奋斗优良传统，反对铺张浪费、奢靡之风，争做清廉型干部，创建节约型机关。

　　各位代表，站在新起点，实现新发展，我们深感使命光荣、责任重大，更感到前景美好、信心倍增。让我们在中共济宁市委的正确领导下，凝聚800万人民的智慧和力量，振奋精神，团结拼搏，开拓创新，真抓实干，全面完成今年各项任务目标，以又好又快发展的新成绩，迎接党的十七大胜利召开！
